# The development of the neurosurgery workforce in Austria over the past quarter century: is more always better?

**DOI:** 10.3389/fmed.2025.1711856

**Published:** 2025-12-03

**Authors:** Mario Mischkulnig, Roland Polacsek-Ernst, Lisa I. Körner, Josa Frischer, Cornelia M. Diendorfer, Christian Matula, Karl Roessler, Georg Widhalm, Christian Dorfer

**Affiliations:** 1 Department of Neurosurgery, Medical University Vienna, Vienna, Austria; 2 University of Applied Sciences BFI Vienna, Vienna, Austria; 3 Department of Management, Economics and Society, Witten/Herdecke University, Witten, Germany; 4 Department of Psychiatry, Medical University Vienna, Vienna, Austria

**Keywords:** neurosurgery, workforce, per capita, density, case numbers

## Abstract

**Background:**

The neurosurgical workforce has expanded markedly across Europe, often accompanied by declining operative exposure per surgeon. Austria, with one of the highest physician and hospital bed densities in the OECD, provides an important case study to assess whether workforce expansion has translated into proportional service provision and maintained training opportunities.

**Methods:**

We performed a retrospective, nationwide analysis of official health statistics from Statistik Austria covering 1997–2023. Data included numbers of practicing neurosurgeons, all specialist physicians, population counts, neurosurgical beds, inpatient stays, and cranial procedures. Absolute and per-capita developments were assessed, and services were related to neurosurgeon density. Statistical analyses comprised Kendall’s tau-b, Wilcoxon signed-rank, and Friedman tests.

**Results:**

The number of practicing neurosurgeons in Austria increased from 97 in 1997 to 301 in 2023 (+ 210.3%), rising from 1.22 to 3.30 per 100,000 inhabitants (+ 170.5%). Growth in neurosurgeon density significantly outpaced both population growth (+ 14.3%) and the overall increase of specialist physicians (+ 77.4%, *p* = 0.001). Despite this expansion, absolute service provision showed only negligible to moderate increases (beds + 4.7%, inpatient stays + 28.6%, cranial procedures + 0.1%). Adjusted for workforce size, services per neurosurgeon declined sharply: cranial procedures decreased by –67.8%, inpatient stays by –58.6%, and neurosurgical bed capacity per surgeon by –66.3% (all *p* < 0.001). Regional disparities were pronounced, with Salzburg reaching 6.51 neurosurgeons per 100,000 while Burgenland registered its first only in 2012 and still shows the nationwide lowest density of 1.00 per 100,000.

**Conclusion:**

Austria has experienced rapid workforce growth without a parallel rise in neurosurgical case volume, resulting in declining operative exposure per surgeon. These findings highlight risks for training quality, efficiency, and future competitiveness. Evidence-based workforce planning, structured regulation of training intake, and expansion of outpatient neurosurgical services will be essential to ensure sustainable care and safeguard international standards of neurosurgical education.

## Introduction

Neurosurgery has evolved into a highly specialized field of medicine in which the availability and distribution of workforce capacity shows substantial regional differences and critically influence patient care (1). Over the past two decades, many European countries have reported a steady rise in the number of practicing neurosurgeons, often accompanied by a substantial decline in procedures performed per surgeon (2, 3). This development has been attributed, among other factors, to the implementation of the European Working Time Directive, which reduced weekly working hours from levels historically exceeding 90–100 h per week to a permissible maximum of 48 h (4–6). While these regulations were intended to improve working conditions and patient safety, there are also concerns that such restrictions impede training opportunities, operative proficiency, and long-term sustainability of service provision, especially in operatively demanding specialties such as neurosurgery (7–9).

Empirical data from neighboring countries support these concerns. In this sense, Ringel et al. found that the number of practicing neurosurgeons in Germany nearly doubled between 2000 and 2019, yet the average annual number of procedures per neurosurgeon declined to 98, of which only 30 were cranial operations (2). Similarly, a European survey from Stienen et al. among residents reported a continuous decrease in operative exposure since the 1960s, with trainees performing on average six fewer procedures per year with each passing decade (8). On a global level, the neurosurgical workforce demonstrates wide international variation, ranging from 0.45 to 2.94 neurosurgeons per 100,000 inhabitants in Europe, with Japan reaching the worldwide highest reported density of 5.6 per 100,000 (2, 10).

Austria occupies a distinctive position in this context. With approximately 540 physicians per 100,000 inhabitants, the country ranks among the highest within the OECD (average 370 per 100,000) (11). Hospital-based care is traditionally extensive, with 690 hospital beds per 100,000 inhabitants compared to an OECD average of 430, and 20,900 annual inpatient stays per 100,000 compared to an OECD average of 13,000 (12). Within neurosurgery specifically, around 5.2 neurosurgical beds per 100,000 inhabitants are available, serving approximately 253 inpatient neurosurgical cases annually per 100,000 inhabitants according to current national health statistics (13). Nonetheless, the neurosurgical outpatient sector (within the framework of Austria’s extensive public health system covering 99.9% of the population) remains primarily based in hospital outpatient clinics, while only three neurosurgical offices hold contracts with public health insurance (12, 14).

In contrast to the already available international evidence of rising neurosurgical workforce numbers accompanied by declining operative volumes per surgeon, it remains unclear whether or to which extent Austria exhibits similar trends. In particular, the extent to which neurosurgical workforce growth has outpaced the physician workforce overall, how regional disparities affect access to care, and how changes in workforce numbers relate to service provision are not yet available in the current literature.

The aim of the present study was therefore to analyze the development of the Austrian neurosurgical workforce over the past quarter century in relation to demographic growth and the physician workforce overall, evaluate regional differences across states, and set it into relation to the provision of neurosurgical services including cranial procedures.

## Materials and methods

In order to investigate the development of the number of practicing neurosurgeons in Austria in relation to the general population and the total number of practicing physicians, as well as to examine regional differences between states and their relation to neurosurgical services provided, we performed a comprehensive analysis of official Austrian data from 1997 to 2023. This period was chosen as it represents the first availability of comprehensive, high-quality data up to the most recent reporting year. All data were collected and made publicly available by Statistik Austria, the federal provider of statistical information including demography and health metrics in Austria. This study was submitted to the ethics committee of the Medical University of Vienna (EK2076/2024), which confirmed that no formal ethics approval was required given the study design without direct patient involvement.

### Data sources

Data on the practicing neurosurgical workforce, physicians of all specialties, neurosurgical services, and demographic parameters for each Austrian state were obtained from *Statistik Austria* (13, 15–18). Since the available information on surgical procedures is categorized by procedure type rather than the specialty of the performing surgeon, we restricted the analysis to cranial procedures in order to avoid the inherent bias that would arise from including procedures such as spine surgery, which are routinely performed also by other specialties. To provide further context, we additionally analyzed the discrepancies between planned (“systematized”) and actually available neurosurgical beds, the development of the average length of stay (ALOS) of neurosurgical patients, and the resulting annual days of neurosurgical bed occupancy. While most data could be retrieved directly from freely available *Statistik Austria* resources, older data were obtained upon request in the form of statistical reports and annual health care yearbooks. Because official statistics until 2006 included dentists in the total physician count, the number of practicing dentists was subtracted for the years 1997–2006 to ensure consistency across the entire observation period. Ultimately, all annual health care yearbooks for Austria from 1997 to 2023 were available, and all variables were retrieved without missing values.

### Statistical methods

The statistical analyses and figures in this study were generated using SPSS Statistics (Version 28.0, IBM Inc., Armonk, NY, United States). Aggregated data were descriptively summarized and visualized with bar charts and combined bar/line diagrams. To examine temporal developments in the number of practicing neurosurgeons and in neurosurgical services, inferential analyses were performed using Kendall’s tau-b test. For comparisons between the development of practicing neurosurgeons and physicians overall, as well as for comparisons of practicing neurosurgeons across Austrian states, the Shapiro–Wilk test indicated non-normal distribution in all groups. Accordingly, the Wilcoxon signed-rank test was applied for the comparison between neurosurgeons and physicians overall, while the Friedman test was used for comparisons across states. The significance level for all analyses was set at *p* < 0.05. Reported *p*-values represent uncorrected results; however, given the consistently very low *p*-values observed (*p* < 0.001), all significant associations would remain robust after Bonferroni–Holm correction for multiple testing.

## Results

Throughout the analyzed period from 1997 to 2023, the number of practicing neurosurgeons in Austria increased from 97 to 301 (+ 210.3%), while at the same time the Austrian population only increased by 14.3% from 7,974,381 to 9,114,200 and the overall number of specialist physicians practicing in any specialty increased from 29,101 to 51,631 (+ 77.4%). All data investigated in this study including the raw data retrieved from Statistik Austria as well variables calculated for the performed analyses are provided in Supplementary Table 1.

### Practicing neurosurgeons per capita

**FIGURE 1:**
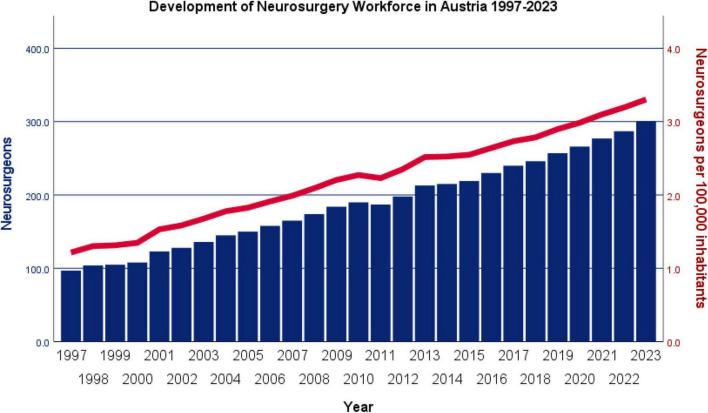
Development of the neurosurgical workforce in Austria between 2000 and 2023 shown as absolute numbers and per 100,000 inhabitants. The number of practicing neurosurgeons in Austria increased steadily from 97 in 2000 to 301 in 2023, corresponding to a rise from 1.22 to 3.30 per 100,000 inhabitants (+ 170.5%).

Corrected for overall population, the number of practicing neurosurgeons in Austria increased from 1.22 per 100,000 inhabitants in 2000 to 3.30 per 100,000 (+ 170.5%) in 2023. This corresponds to an average annual growth rate of 4.0% in the number of practicing neurosurgeons per 100,000 inhabitants, with a strictly monotonic increase observed in all years except 2011, when a slight decrease from 2.28 to 2.23 per 100,000 was seen. Inferential statistical analysis using the Kendall’s tau-b test confirmed a highly significant upward trend (τ = 0.99, *p* < 0.001) in the number of practicing neurosurgeons per 100,000 inhabitants between 2000 and 2021. A bar chart illustrating the increase in the neurosurgical workforce in Austria, both in absolute numbers and relative to population size, is presented in Figure 1.

### Comparison with physicians of all specialties

**FIGURE 2:**
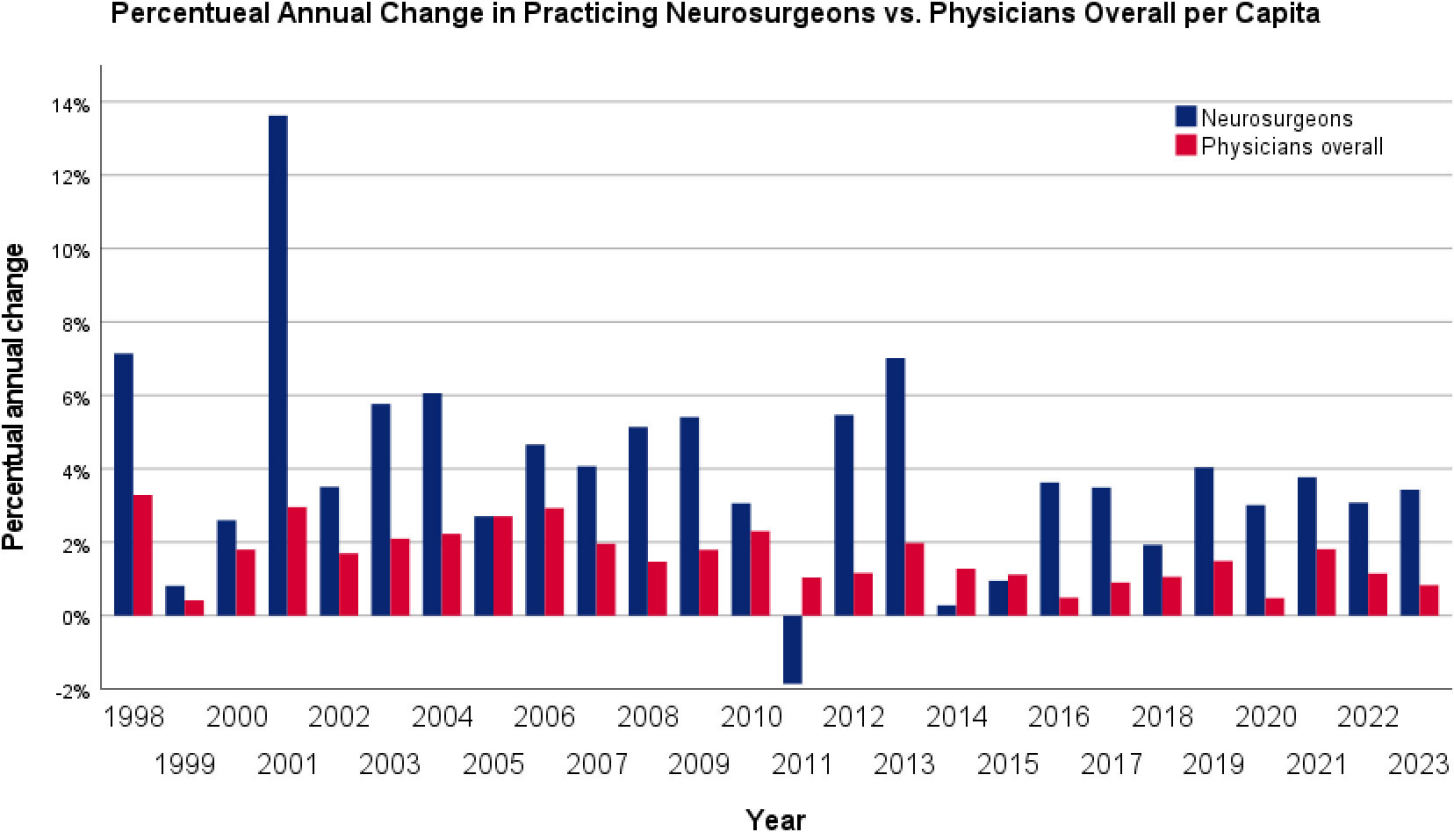
Annual percentage change in the number of practicing neurosurgeons compared with all physicians in Austria. While the number of physicians overall grew by an average of 1.6% per year, neurosurgeons showed a significantly higher average annual increase of 4.0% (*p* = 0.001, Wilcoxon signed-rank test).

Corrected for demographic growth, the number of practicing physicians of all specialties increased from 364.93 per 100,000 inhabitants in 1997 to 555.52 per 100,000 in 2023, corresponding to an average annual growth rate of 1.6%. Statistical analysis using the Wilcoxon signed-rank test demonstrated a significantly higher average annual increase among practicing neurosurgeons compared to physicians overall (*p* = 0.001) during the study period. The annual percentage change in the number of practicing neurosurgeons and physicians overall per 100,000 inhabitants is illustrated in Figure 2. In addition to the 2.5-fold higher average annual increase among neurosurgeons compared to physicians overall, it is noteworthy that particularly large differences in growth rates were observed in the early years of the observation period. These early pronounced differences resulted in even higher effects between both groups throughout the entire period due to compounding effects of early differences than considered in this statistical model.

### Regional distribution

**FIGURE 3:**
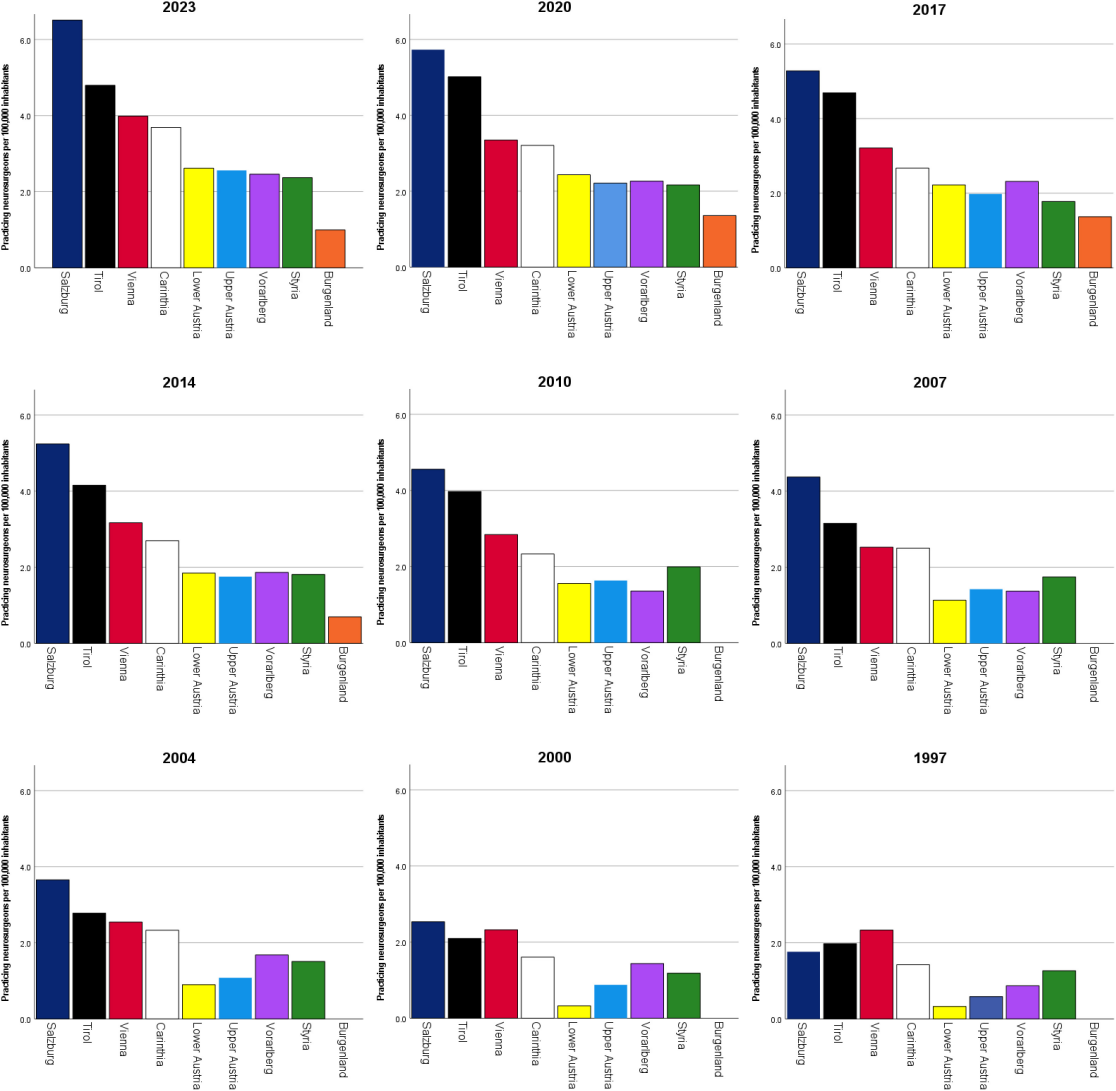
Regional development of practicing neurosurgeons per 100,000 inhabitants in the nine Austrian states (1997–2023). Substantial regional disparities were observed (*p* < 0.001, Friedman test). Salzburg consistently showed the highest densities (up to 6.51 per 100,000 in 2023), while Burgenland remained lowest, only registering its first neurosurgeon in 2012.

While increases in the number of practicing neurosurgeons per 100,000 inhabitants between 2000 and 2021 were observed across all nine Austrian states, highly statistically significant regional differences were evident (*p* < 0.001). Salzburg consistently showed the highest numbers in all but the first 2 years of observation, with an increase from 1.76 to 6.51 per 100,000 (+ 269.8%). In contrast, no practicing neurosurgeon was registered in Burgenland until 2012. However, by 2016 the number had risen to 1.37 per 100,000. Although this remained the lowest rate compared to other states, it already exceeded the national average observed in 2000. Statistical analysis using the Friedman test confirmed a highly significant difference in the number of practicing neurosurgeons per 100,000 inhabitants across Austrian states (*p* < 0.001). Figure 3 illustrates the temporal development of practicing neurosurgeons per 100,000 inhabitants in the nine Austrian states between 1997 and 2023, and the underlying yearly data are provided in Table 1.

**TABLE 1:** Practicing neurosurgeons per 100,000 inhabitants in Austria by state between 1997 and 2023.

Year	Salzburg	Tirol	Vienna	Carinthia	Lower Austria	Upper Austria	Vorarlberg	Styria	Burgenland
**Practicing neurosurgeons per 100,000 inhabitants**									
2023	6.51	4.80	3.99	3.69	2.62	2.56	2.46	2.37	1.00
2022	6.04	5.37	3.68	3.37	2.59	2.33	2.49	2.31	1.34
2021	6.24	5.26	3.44	3.38	2.42	2.27	2.50	2.25	1.35
2020	5.73	5.02	3.35	3.21	2.43	2.21	2.27	2.17	1.36
2019	5.40	4.77	3.21	3.03	2.32	2.16	2.54	2.25	1.36
2018	5.07	4.66	3.28	2.67	2.09	2.17	2.30	2.10	1.37
2017	5.28	4.69	3.21	2.67	2.22	1.98	2.32	1.78	1.37
2016	5.31	4.33	3.26	2.50	1.94	1.93	2.34	1.79	1.37
2015	5.38	4.12	3.12	2.51	1.89	1.74	2.38	1.88	0.69
2014	5.24	4.15	3.17	2.70	1.85	1.75	1.87	1.81	0.70
2013	5.08	3.91	3.10	2.70	1.67	1.90	2.42	1.98	0.70
2012	4.91	3.23	3.14	1.98	1.61	1.84	1.35	1.90	0.35
2011	4.55	3.53	2.88	2.16	1.55	1.70	1.08	1.99	0.00
2010	4.56	3.97	2.84	2.33	1.56	1.63	1.36	1.99	0.00
2009	4.56	3.42	2.68	2.50	1.50	1.63	1.91	1.91	0.00
2008	4.56	3.14	2.63	2.50	1.38	1.56	1.09	1.83	0.00
2007	4.37	3.16	2.53	2.50	1.13	1.42	1.37	1.75	0.00
2006	4.19	2.74	2.66	2.32	1.20	1.14	1.38	1.67	0.00
2005	3.83	2.90	2.57	2.33	0.89	1.15	1.39	1.67	0.00
2004	3.66	2.78	2.55	2.33	0.90	1.08	1.68	1.51	0.00
2003	3.29	2.50	2.51	2.15	0.84	1.01	1.69	1.43	0.00
2002	2.71	2.37	2.61	1.61	0.71	0.94	1.70	1.52	0.00
2001	2.72	2.53	2.57	1.61	0.52	0.87	1.43	1.52	0.00
2000	2.53	2.10	2.32	1.61	0.33	0.88	1.44	1.18	0.00
1999	2.93	1.81	2.27	1.60	0.26	0.88	1.15	1.18	0.00
1998	2.15	1.82	2.47	1.43	0.39	0.81	0.87	1.27	0.00
1997	1.76	1.98	2.33	1.42	0.33	0.59	0.87	1.27	0.00

### Neurosurgical services per neurosurgeon

**FIGURE 4:**
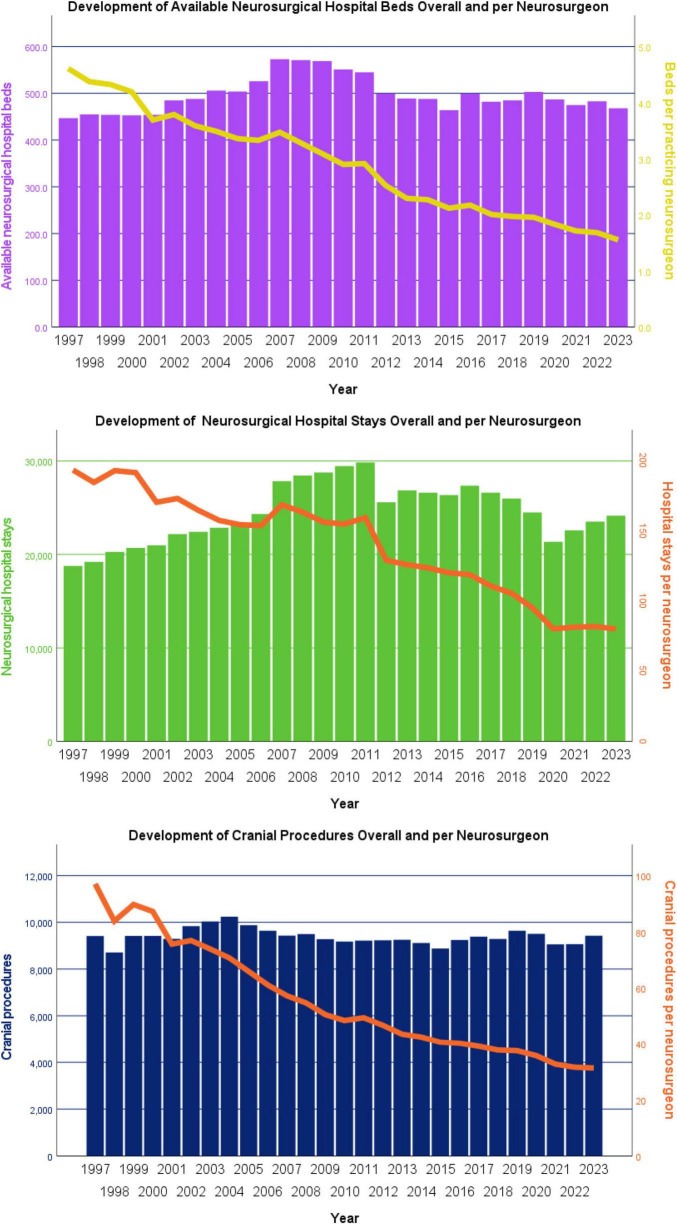
Development of neurosurgical services in Austria (1997–2023), shown as absolute numbers and per practicing neurosurgeon. Despite small to moderate increases in overall services a marked decline per neurosurgeon was observed. Services per neurosurgeon fell by –66.3% for beds, –58.6% for inpatient stays, and –67.8% for cranial procedures (all *p* < 0.001).

In contrast to the marked increase in the number of practicing neurosurgeons over the past 22 years, only a negligible to moderate rise was observed in the overall provision of neurosurgical services, including available hospital beds (+ 4.7%), inpatient stays (+ 28.6%), and cranial procedures (+ 0.1%). When adjusted for the number of practicing neurosurgeons in each year, however, a pronounced decline in services per neurosurgeon became evident. Specifically, the number of available neurosurgical hospital beds per practicing neurosurgeon significantly decreased from 4.60 in 1997 to 1.55 (–66.3%) in 2023 (τ = –0.972, *p* < 0.001). Similarly, annual inpatient hospital stays per neurosurgeon declined significantly from 193.5 in 1997 to 80.2 (–58.6%) in 2023 (τ = –0.875, *p* < 0.001). Finally, the average annual number of cranial procedures per neurosurgeon showed a significant reduction from 97.1 in 1997 to 31.3 (–67.8%) in 2023 (τ = –0.977, *p* < 0.001). A graphical representation of the overall neurosurgical services and their per-neurosurgeon distribution is presented in Figure 4.

### Hospital bed capacity and ALOS trends

**FIGURE 5:**
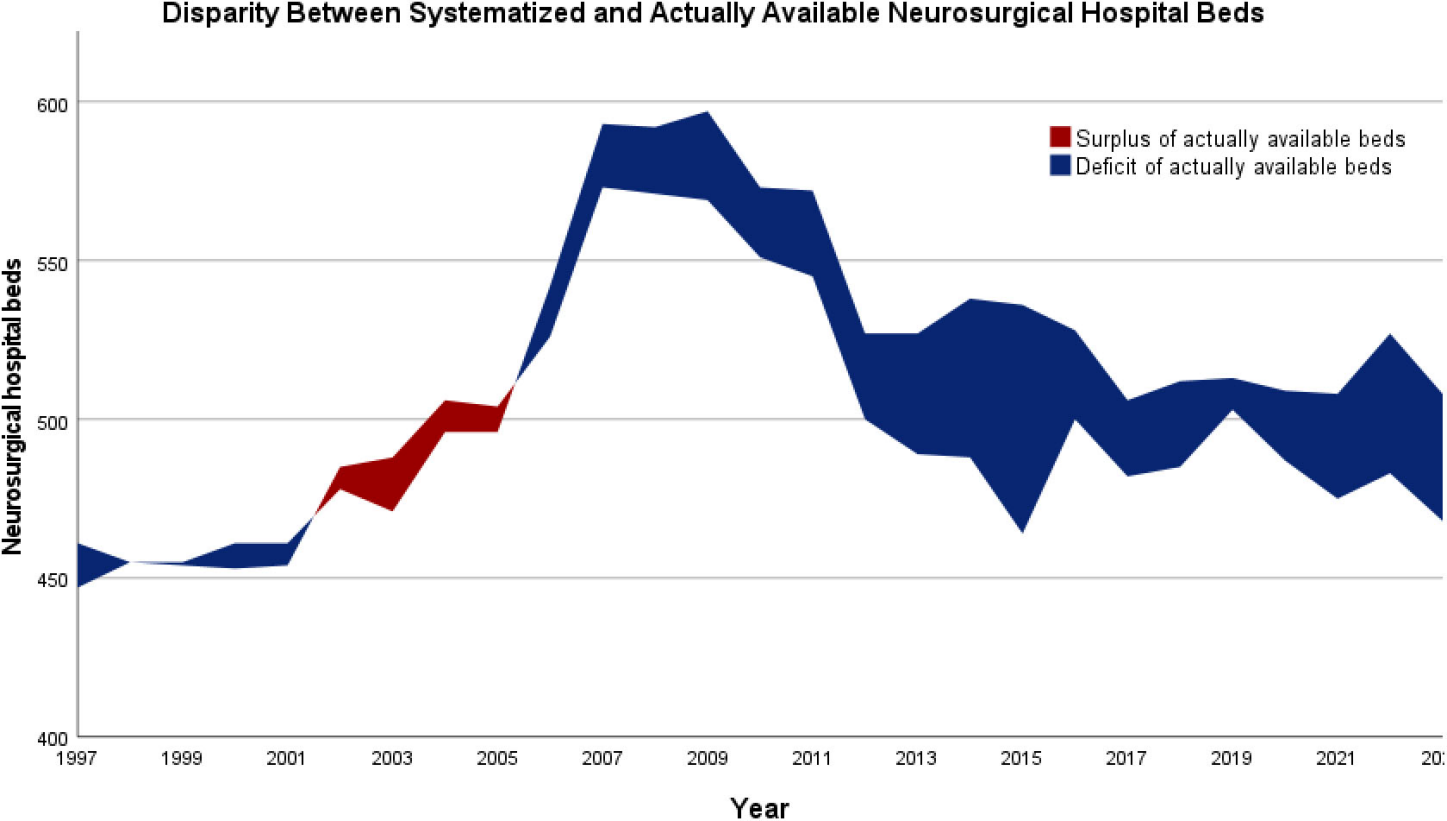
Disparity between systematized (planned) and actually available neurosurgical hospital beds in Austria (1997–2023). A persistent deficit of actually available neurosurgical beds has been present since 2006, peaking at –72 beds in 2015 and remaining at –40 beds in 2023. Small surpluses compared to planned beds were only observed between 2002 and 2005.

To gain further insights into factors contributing to the substantial decrease in neurosurgical services per practicing neurosurgeon, we first examined the discrepancy between the systematized (planned) and the actually available neurosurgical hospital beds for each year. This analysis revealed a small deficit of up to 14 beds between 1997 and 2001, followed by a brief period of surplus of up to 17 beds between 2002 and 2005. Since 2006, however, there has been a persistent deficit of actually available neurosurgical hospital beds, peaking at 72 beds in 2015 and still amounting to 40 beds in the most recent data from 2023. The disparity between systematized and actually available neurosurgical hospital beds in Austria between 1997 and 2023 is illustrated in Figure 5.

**FIGURE 6:**
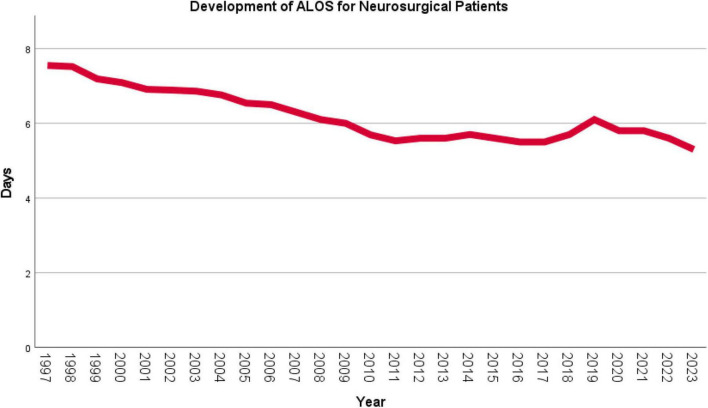
Development of the average length of stay (ALOS) of neurosurgical inpatients in Austria (1997–2023). The mean ALOS of neurosurgical patients decreased significantly from 7.6 days in 1997 to 5.3 days in 2023 (–30.3%; *p* < 0.001).

With respect to the average length of stay (ALOS) of neurosurgical patients, a continuous and highly statistically significant decrease was observed (τ = –0.726, *p* < 0.001) from 7.6 days in 1997 to 5.3 days in 2023 (–30.3%), as shown in Figure 6.

**FIGURE 7:**
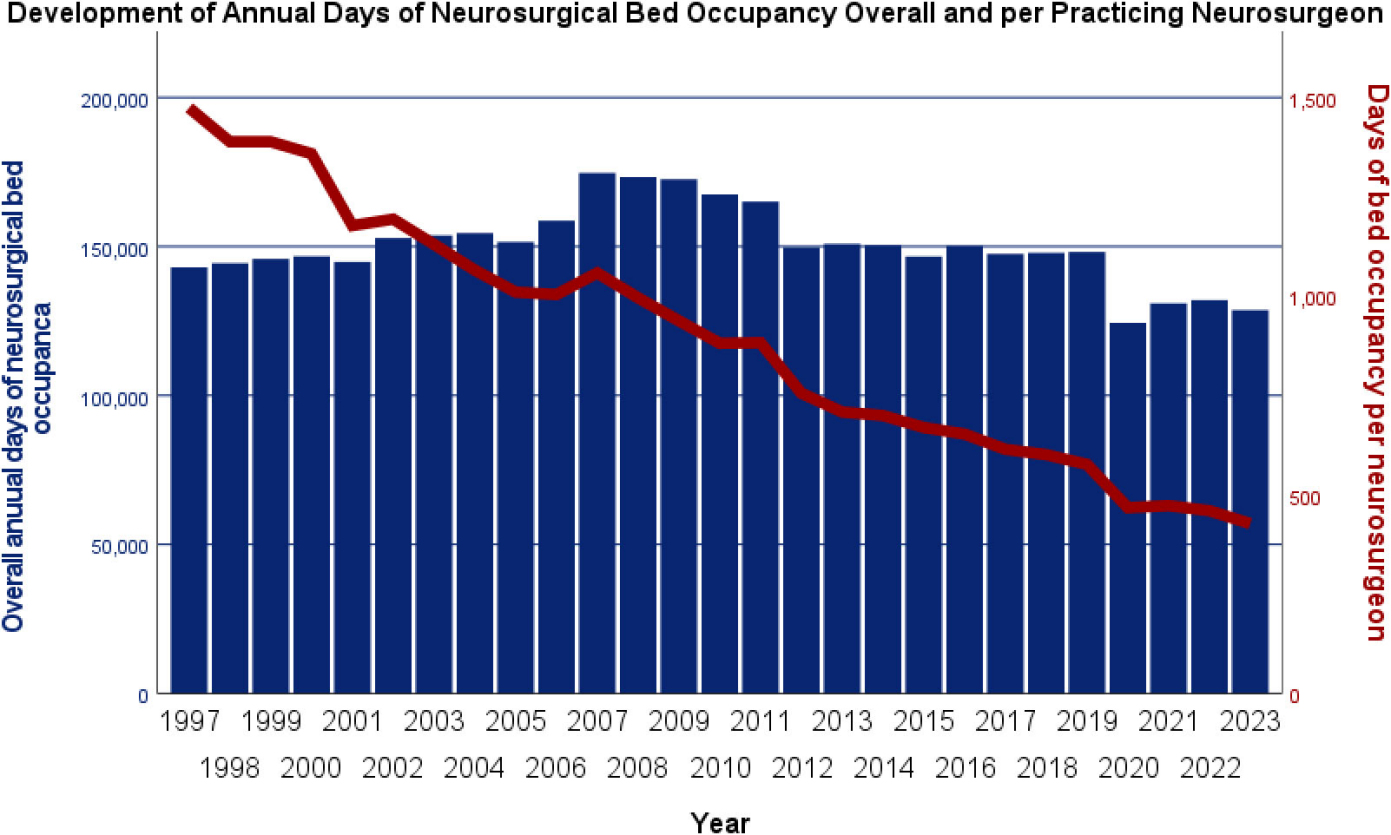
Annual days of neurosurgical bed occupancy in Austria (1997–2023), shown overall and per practicing neurosurgeon. Overall neurosurgical bed occupancy decreased slightly from 143,000 days in 1997 to 128,708 days in 2023 (–10.0%; not significant). Per neurosurgeon, however, bed-days dropped markedly from 1,474.2 to 427.6 (–71.0%; *p* < 0.001).

In the subsequent analysis of annual neurosurgical days of neurosurgical bed occupancy, reflecting both the moderate increase in available hospital beds and the marked reduction in ALOS, a slight but not statistically significant decline was found (τ = –0.185, *p* = 0.295), from 143,000 in 1997 to 128,708 in 2023 (–10.0%). When expressed per practicing neurosurgeon, however, a highly significant decrease became evident (τ = –0.972, *p* < 0.001), from 1,474.2 days in 1997 to 427.6 days (–71.0%) in 2023. The development of total and per-neurosurgeon annual neurosurgical bed-days in Austria is presented in Figure 7.

## Discussion

In this study, we document a marked expansion of Austria’s neurosurgical workforce over the past decades, which has far outpaced population growth and even the overall rise in physicians. In contrast, absolute neurosurgical case volumes have not increased in parallel, and when related to the growing number of practicing neurosurgeons, a clear decline in neurosurgical services in general and cranial procedures in particular per surgeon becomes evident. These findings raise important concerns about training quality, efficiency, and future competitiveness.

### Policy changes and systemic constraints

Two major policy levers have shaped Austria’s neurosurgical service environment: the LKF (Leistungsorientierte Krankenhausfinanzierung) system, introduced in 1997, and the implementation of the European Working Time Directive (WTD) into Austrian law (KA-AZG) by 2015 (6, 19, 20). The LKF was intended to reduce the average length of stay (ALOS) and decrease bed capacity by linking hospital funding more strictly to performance metrics. Consistent with that aim, Austria has achieved reductions in ALOS over time, though it remains among the highest in the OECD (12, 20). However, lowering LOS does not automatically reduce inpatient demand, nor does it necessarily shift enough activity into the outpatient sector without structural reforms (7, 21).

The WTD has imposed a cap of 48 h/week on physician working time, with the aim of improving work-life balance and preventing fatigue. Survey data confirm that working hours in Austria and across Europe have been substantially curtailed (19, 22). At the same time, even working hour restrictions of 80 h imposed on residents in the United States have led to concerns regarding a negative impact of training quality and neurosurgery programs are therefore particularly often exempt from these limitations (9, 23). Meanwhile there are reports available that indicate unintended consequences of the European WTD in form of reduced operative exposure during residency, fragmentation of care, and increased workforce demand to cover the same service load (2, 8, 24, 25).

### Training volume, exposure, and competitiveness

One major implication of these systemic reforms is reduced procedural volume per neurosurgeon. Across Europe, neurosurgical residents report a steady decline in case numbers over the past decades, averaging six fewer operations annually with each passing decade (8). In Germany, where the number of practicing neurosurgeons more than doubled between 2000 and 2020, the average annual case load per surgeon fell to below 100 operations, of which only about 30 were cranial procedures (2). These trends parallel the Austrian experience documented in this study and underscore the risk of dilution of training quality. One possible solution in order to deal with decreasing overall procedure numbers is an increased amount of subspecialization that has been taking place over the past years not just in neurosurgery but a variety of surgical specializes (26). In this sense, the expansion of the available workforce may not only require but in fact also facilitate the increasing subspecialization within neurosurgery, allowing neurosurgeons to focus more narrowly on defined fields and thus further advance specific surgical skills.

Unlike in the United States, where neurosurgical training is governed by a nationally standardized curriculum with centralized oversight and clearly enforced operative volume thresholds (27), Austria’s system is more decentralized. Although minimum operative requirements exist (28), their monitoring and enforcement remain less systematic, and there is no central authority that regulates training intake or aligns trainee numbers with projected workforce needs. Furthermore, Austria, like other European countries, lacks a functioning incentive structure that ties training quality and operative exposure to institutional accreditation or funding, as is common in the United States system (2, 8). At the European level, frameworks such as the EANS curriculum and the European Board examinations aim to harmonize training standards, yet participation so far remains voluntary and does not replace binding national oversight (29). This relative absence of centralized governance and incentives risks creating variability in training quality and may ultimately affect the international competitiveness of Austrian neurosurgeons.

### Outpatient care and service delivery gaps

Despite reductions in LOS and hospital utilization, outpatient neurosurgical care remains underdeveloped in Austria. Of nearly 150 practicing neurosurgeons in private practice, only three currently hold contracts with public health insurance (14). This forces patients to rely heavily on hospital-based care for even routine follow-up or minor interventions, limiting continuity of care and inflating hospital resource utilization. By contrast, countries with stronger outpatient neurosurgical sectors, such as the United States and parts of Scandinavia, have demonstrated that shifting appropriate cases to ambulatory settings can improve cost-effectiveness and efficiency (7, 10).

### Workforce regulation and planning

Austria currently has no authority regulating neurosurgeon training intake. In contrast, systems such as the UK and US apply strict controls over residency slots and accreditation, aligning training numbers with projected service needs (30, 31). Without such regulation, oversupply relative to procedural volume risks further reducing case exposure per trainee and diluting expertise. Regional maldistribution adds to the challenge: Salzburg reached densities above 6.5 neurosurgeons per 100,000 inhabitants in 2023, while Burgenland only registered its first neurosurgeon in 2012. This uneven distribution may lead to inequities in timely access, even within a small and well-insured country.

### Economic and systemic trade-offs

Workforce expansion without proportional service volume has cost implications. Austria already has one of the highest physician and hospital bed densities in the OECD (12). Adding more neurosurgeons without increased throughput risks driving up per-case costs. Additionally, the increased expense of training programs under reduced working-hour conditions may strain both institutions and trainees. International analyses in surgical education have emphasized that working time restrictions, without compensatory measures such as simulation or team restructuring, threaten to compromise both training quality and efficiency (23, 32, 33).

Nevertheless, increases in workforce undoubtedly also offer opportunities and potential benefits for the output of neurosurgical services. Besides a reserve for routine patient care provided that minimum requirements for surgical training and practice are sustained, this applies in particular to further core functions of modern neurosurgy such as medical teaching and research. In this sense, the number of Pubmed listed publications retrieved with the query “neurosurgery austria” increased since the beginning of the observation period of this study in 1997 from 39 to 508 in 2024 (34). Likewise, according to data published in the last available performance report of the Department of Neurosurgery at the Medical University of Vienna, teaching activities more than tripled in the years leading up to the last analyzed academic year 2020/21 (35). Along with novel *ex vivo* training techniques that are already being implemented in neurosurgical education, these findings provide an upside to the substantial challenges posed by the reported workforce data (36, 37).

### Limitations

Several limitations of this study should be acknowledged. First, the analyses relied on aggregated national health statistics, which, although comprehensive, do not allow differentiation by case complexity or individual surgeon activity. As such, reductions in procedures per surgeon may partly reflect a shift toward more complex cases rather than reduced overall expertise. Second, the observational design does not allow causal inference; policy changes such as the introduction of the LKF system or working time restrictions coincided with, but cannot be definitively linked to, the observed trends. Finally, international comparisons must be interpreted with caution, as differences in coding systems, health care structures, and training pathways may limit direct comparability.

### Recommendations and outlook

To preserve the quality of neurosurgical care and training in Austria, several measures should be considered:

1. Evidence-based workforce planning to align training intake with projected demand on a national and regional level.
2. Implementation of a structured national residency allocation with limited and competitively assigned training slots based operative volume and training quality.
3. Expansion of outpatient neurosurgical care, including broader insurance coverage for office-based neurosurgeons.
4. Compensatory training strategies such as surgical simulation, skills labs, and redistribution of administrative tasks to allied health professionals in order to ascertain sufficient surgical skill development and maintenance despite challenges posed by work time restrictions.
5. Adoption of Europe-wide board certification standards and continuous monitoring of training outcomes to ensure sustainable international competitiveness.

## Conclusion

Austria has witnessed a rapid expansion of its neurosurgical workforce over the past quarter century, outpacing both demographic growth and the increase of physicians overall. However, this growth has not been matched by higher case volumes, leading to a marked decline in procedures and services per surgeon. These findings highlight the need for evidence-based workforce planning, structured training regulation, and expansion of outpatient care to ensure sustainable service provision and to safeguard training quality and international competitiveness.

## Data Availability

The original contributions presented in this study are included in this article/Supplementary material, further inquiries can be directed to the corresponding author.
